# Beliefs and knowledge about post-traumatic stress disorder amongst resettled Afghan refugees in Australia

**DOI:** 10.1186/s13033-016-0065-7

**Published:** 2016-04-12

**Authors:** Anisa Yaser, Shameran Slewa-Younan, Caroline A. Smith, Rebecca E. Olson, Maria Gabriela Uribe Guajardo, Jonathan Mond

**Affiliations:** Mental Health, Centre for Health Research, School of Medicine, Western Sydney University, Sydney, Australia; National Institute of Complementary Medicine, Western Sydney University, Sydney, Australia; School of Social Science, Faculty of Humanities and Social Sciences, University of Queensland, Brisbane, Australia; Department of Psychology, Macquarie University, Sydney, Australia

**Keywords:** Mental health literacy, Post-traumatic stress disorder, Afghan refugees

## Abstract

**Background:**

Resettled refugees are at high risk of trauma-related mental health problems, yet there is low uptake of mental health care in this population. Evidence suggests poor ‘mental health literacy’ (MHL) may be a major factor influencing help-seeking behaviour among individuals with mental health problems. This study sought to examine the MHL of resettled Afghan refugees in Adelaide, South Australia.

**Methods:**

Interviews were completed with 150 (74 males; mean age 32.8 years, SD = 12.2) resettled Afghan refugees living in Adelaide, South Australia. A convenience sampling method was employed and participants were comprised of volunteers from the Afghan community residing in the northern suburbs of Adelaide. Following informed consent participants were presented a culturally appropriate vignette describing a fictional person suffering from posttraumatic stress disorder (PTSD). This was followed by a series of questions addressing participants’ knowledge and understanding of the nature and treatment of the problem described. Self-report measures of PTSD symptoms and co-morbid psychopathology were also administered.

**Results:**

Thirty-one per cent of the respondents identified the problem depicted in the vignette as being PTSD, while 26 per cent believed that the main problem was ‘fear’. Eighteen per cent of participants believed that ‘getting out and about more/finding some new hobbies’ would be the most helpful form of treatment for the problem described, followed by ‘improving their diet’ and ‘getting more exercise’ (16 %).

**Conclusion:**

The results of this study demonstrate aspects of MHL that appear to be specific to Afghan refugees who have resettled in Australia. They indicate the need for health promotion and early intervention programs, and mental health services, to recognise that variation in MHL may be a function of both the cultural origin of a refugee population and their resettlement country. Such recognition is needed in order to bridge the gap between Western, biomedical models for mental health care and the knowledge and beliefs of resettled refugee populations.

**Electronic supplementary material:**

The online version of this article (doi:10.1186/s13033-016-0065-7) contains supplementary material, which is available to authorized users.

## Background

Over the past two decades there has been an increased interest in research examining the mental health outcomes of refugees because of their exposure to potentially traumatic events (PTE’s) both prior and subsequent to their displacement from their homelands [1]. Common refugee experiences include torture, war or civil unrest, the loss of family and friends through violence, and prolonged periods of deprivation. Exposure to these adverse events can have a significant impact on psychological health and wellbeing, most commonly manifested by the psychiatric conditions of post-traumatic stress disorder (PTSD) and depression [2–4].

Research in this field is now seeking to move beyond documenting the impact that trauma exposure has on mental health outcomes in refugees, with an increased number of studies seeking to inform best practice treatment, early intervention and health promotion strategies for these populations [5]. This is based on the knowledge that factors such as cultural differences can influence the acceptability and efficacy of such interventions [6]. Indeed, it has been consistently documented that in spite of the high prevalence of mental health disorders in refugee groups [7], their mental health utilisation is well below that of the general population [8]. A lack of understanding of what constitutes mental illness may be a barrier to the uptake of mental health care, due in part to differences in cultural perceptions of mental health [9]. For example, evidence suggests that Muslim refugees tend to hold differing views of mental health and that this may be associated with reduced utilisation of these services [10, 11]. Thus, elucidating refugees’ awareness and understanding of mental health problems and their treatment has the potential to improve the uptake of mental health care where this is needed and inform the development of health promotion and early intervention programs.

Mental Health Literacy (MHL) refers to, ‘knowledge and beliefs about mental disorders which aid their recognition, management or prevention’ [12, p. 183]. This encompasses (a) the ability to recognize specific disorders; (b) knowledge of how to seek mental health information; (c) knowledge of risk factors and causes, of self-treatments, and of professional help available; and (d) attitudes that promotes recognition and appropriate help-seeking [12]. Improving MHL aims to: (i) empower the public with an understanding of mental health disorders, thereby facilitating the implementation of prevention, early intervention and treatment programs [13]; and (ii) empower individuals in need of mental health care with the means by which to make an informed decision about accessing this care.

Since the seminal paper in 1997 [12], there have been multiple national surveys of MHL conducted with the general Australia population [14, 15], demonstrating the under development of public knowledge and the need for targeted mental health education and promotion campaigns [13]. By comparison, the MHL of specific communities such as resettled refugees remains far less investigated [13]. It is acknowledged that given the diversity and heterogeneous nature of this community, which originates from a range of cultures, nationalities and religions, studies of refugee mental health are best undertaken specific to a refugee subgroup. Thus, a recent study of MHL relating to PTSD among resettled Iraqi refugees in Australia [16] found that individuals in this community had low levels of problem recognition (<15 % correctly identified PTSD when presented with a vignette of this condition) and diverse beliefs concerning treatment options including preferences for both Western biomedical approaches and reading religious texts [16].

Data from the Australian Department of Immigration and Border Protection (DIBP) notes that since 2010 the highest proportion of those deemed ‘irregular maritime arrivals’, more commonly known as asylum seekers, who subsequently apply for onshore protection are those that originate from Afghanistan [17]. Further, there is a small but growing literature documenting poor physical and mental health outcomes of resettled Afghan refugees [18]. Indeed, in a systematic review that included studies conducted with Afghan refugees resettled in Western nations, it was reported that depression and PTSD prevalence rates were as high as 57 and 100 %, respectively [4].

Taken together, these considerations suggest that research addressing the MHL of these individuals relating to PTSD would also be of interest. The goal of the current study was, therefore, to examine knowledge of, and beliefs about, the treatment of and help-seeking for PTSD amongst Afghan refugees resettled in Australia. We were also interested to consider the associations between participants’ socio-demographic characteristics, level of PTSD and depression, and responses to particular aspects of MHL.

## Methods

### Study design and participants

Approval for the study was obtained from the relevant university Human Research Ethics Committee (H10048). Efforts were made by the author (AY) to promote the study in the South Australian Afghan community through networking at Afghan cultural, religious and other Afghan gatherings, and by placing flyers (translated into Dari) on the walls of venues (e.g., grocery stores) known to be frequented by the Afghan population in Adelaide, South Australia. The flyers included information concerning the study aims, the time commitment entailed in participation and the inclusion criteria. Prior to the flyers being posted, a meeting was held with community leaders to explain the project and to seek permission to place the flyers on venues deemed appropriate. This permission was granted.

A combination of convenience and snowball sampling was employed to maximise participation. Most participants voluntarily responded by contacting the researcher on the mobile number provided on flyers; a small number of participants were introduced to the research through family or friends, the latter passing contact details onto the researcher. Once participants confirmed their willingness to participate the researcher then followed up by phoning each individual back to explain the aim of the research and set a convenient time for the interview. Interviews were conducted individually and, most often, in the homes of the participants. Several interviews were conducted at the Adelaide and South Australian University libraries as well as public libraries. Two interviews were conducted in researcher AY’s office. Prior to each interview participants were provided with a participant information sheet that briefly outlined the study and aims of the research. Following this they were asked again if they wished to participate in the research project. Written informed consent was then obtained from all participants. Next, a culturally appropriate vignette describing a fictional person suffering from posttraumatic stress disorder (PTSD) was presented, followed by a series of questions addressing participants’ knowledge and understanding of the nature and treatment of the problem described. Self-report measures of PTSD symptoms and co-morbid psychopathology were also administered. The process of data collection was undertaken by author AY (fluent in Dari and English) and each session ranged from 60 to 90 min, with traditional cultural hospitality arrangements, including participants preparing and offering sweets, tea or coffee, accounting for this variation in the length of each interview. Of the total 164 people who initially made contact, 14 declined participation nominating work or family commitments as reasons for non-participation. The inclusion criteria included having been born in Afghanistan, having left Afghanistan during or after 2000, being fluent in Dari and/or English, and being between 18 and 70 years of age. Resettlement during or following 2000 was required in order to establish a more homogeneous sample in terms of exposure to conflict, namely, individuals who were living in Afghanistan following the arrival of the Taliban regime. All participants were provided with (translated) information sheets containing details of local specific mental health services. In appreciation of their time, a food gift voucher in the amount of AUD $25.00 was provided to all participants upon completion of the survey.

### Measures

#### The mental health literacy survey

The survey was modelled on Jorm et al.’s [12] protocol, with modifications by the authors (SSY, AY and JM) for the study of PTSD in refugees. Specifically, the vignette was developed based on the consensus of several authors (SSY and AY) experienced in the assessment and/or clinical treatment of PTSD in refugees. Care was taken to ensure the vignette was culturally appropriate through the use of simple language, respecting cultural sensitivities and assigning common names to the fictional characters in the vignette. The final survey was translated into Dari and independently back-translated into English using a nationally accredited translation and interpreting service. All discrepancies were checked and rectified by the translators and the research team [19]. The vignette (see Additional file 1), which was read aloud by the interviewer, described a fictional Afghan refugee ‘Mariam or Ahmad’ (sex of vignette character was matched to sex of participant being interviewed), who had been exposed to trauma prior to leaving Afghanistan and who was suffering symptoms of PTSD, according to criteria outlined in the 4th edition of the diagnostic and statistical manual of mental disorders (DSM IV-TR, American Psychiatric Association). A prompt card in Dari was provided so that the participant could follow the description as it was read and participants were advised that they could refer back to the vignette at any time during the interview. Following the presentation of the vignette, participants were asked: ‘What would you say is Mariam’s/Ahmad’s main problem’? Participants were required to choose only one answer from a number of options. Listed in random order, these were: ‘fear’; ‘no real problem’; ‘just a phase’; ‘depression’; ‘weak character’; ‘nervous breakdown’; ‘posttraumatic stress disorder’; ‘serious medical condition (e.g., brain tumour)’; ‘stress’; ‘not integrating well in Australia/homesickness’; and ‘physical condition (e.g., migraine or back pain)’. Participants’ beliefs about the helpfulness of various interventions for the problem described in the vignette were also assessed. Specifically, participants were asked whether each of a number of interventions, within the categories of treatment activities, medicines and people, would be helpful, harmful or neither (helpful nor harmful) for the person described in the vignette. The selection of treatment activities, medicines and people in this study were based on a consensus of several authors (SSY and AY) experienced in the assessment and/or clinical treatment of PTSD in refugees and reflective of those offered in the 2011 National Survey of Mental Health Literacy and Stigma (NSMHLS) [15]. Additionally, participants were asked which intervention within each category they believed would be *most* helpful for this person.

#### Exposure to war related violence and loss

Following completion of the MHL survey the Afghan War Experience Scale (AWES) was used [20] to assess exposure to war-related violence and loss. The scale, developed with the Afghan population, asks participants to indicate whether they have experienced the noted 17 war-related experiences of violence or loss with response choices including never (0), once (1), or more that once (2). Scores on the 17 items are totalled, yielding a possible range of 0–34, with higher total scores reflecting greater exposure to war-related experiences. This scale was administered to document levels of exposure to war related violence and loss.

#### PTSD symptoms

The impact of events scale-revised [21] (IES-R; 18), a widely used, self-report scale that assesses PTSD symptomatology, was also adopted in this study. It has strong psychometric properties with internal consistency reported as 0.96 [22]. In a recent study [23], Morina and Colleagues (2013) examined the diagnostic utility of the IES-R in two samples of war affected populations (n = 3313 and n = 854) noting that a score of 34 or above indicates a high probability of clinically significant PTSD symptomatology. Cronbach’s alpha in the current study was 0.965.

#### Self-reported depression symptoms

Severity of depression symptoms was assessed using the Hopkins Symptoms Checklist–25 [24]. The HSCL–25 is a 25-item questionnaire made up of two subscales measuring anxiety symptoms (10 items) and depression symptoms (15 items). The HSCL–25 has been used extensively in refugee populations, including Afghan refugees [18]. For the purposes of this study, only the depression subscale is reported and the noted community cutoff point for the HSCL–25 depression subscale of 1.75 was utilised [24]. The HSCL-25 demonstrates high internal consistency (α = 0.85), high test–retest reliability (*r* = 0.82 for each subscale), and good validity (88 % sensitivity, 73 % specificity) in diagnosing depression [24]. Cronbach’s alpha in the current study was 0.958.

Demographic information obtained included: age in years; sex; arrival status (refugee, asylum seeker, immigrant); length of time (months) in Australia; length of time (months) externally displaced; marital status (never married, married/living as married, separated or divorced, widowed); and years of formal education completed.

### Statistical analyses

With 150 respondents, the study had 80 % power at 5 % significance level to detect a medium effect size of between 0.23 and 0.27 using 1–3 degrees of freedom Chi square test, and an effect size of 0.33 for Mann–Whitney tests of associations between key demographic variables, symptom levels and specific aspects of MHL.

Statistical analysis was carried out using IBM SPSS Statistics version 22.0. The effect of socio-demographic characteristics and symptom levels on the HSCL-25 (depression) and IES-R (as defined in the scale descriptions) on responses regarding problem recognition and beliefs about interventions were examined using Mann–Whitney U tests, Kruskal–Wallis tests, Spearman’s rank correlations or Chi square tests, as appropriate (for the purpose of this analysis, responses to questions concerning the perceived helpfulness of particular interventions were recoded: helpful = 1, harmful = −1, and neither = 0). Pairwise post hoc comparisons of significant socio-demographic characteristics were performed using Dunn’s procedure [25] with a Bonferroni correction for multiple comparisons, with adjusted p values reported. In view of the number of tests conducted, a significance level of 0.010 was employed for all tests. Missing data was low, in the order of <1 % and was handled by listwise deletion. Where appropriate, data for levels of general psychological distress and PTSD symptomatology derived from the Second (2007) Australian National Survey of Mental Health and Wellbeing (NSMHWB) [14] and responses from the 2011 National Survey of Mental Health Literacy and Stigma (NSMHLS) [15] were used for comparative purposes.

## Results

The demographic and clinical characteristics of participants (n = 150) are shown in Table 1.

**Table 1:** Demographic and clinical characteristics of study participants (n = 150)

Characteristics	N (total = 150)	%
Gender		
Male	74	49.3
Female	76	50.7
Age in years, mean (SD)	32.8 (12.3)	
Years of education, mean (SD)	6.1 (5.2)	
Months in Australia, mean (SD)	71.4 (49.6)	
Months externally displaced, mean (SD)	67.5 (68.8)	
Arrival status to Australia		
Refugee	64	42.7
Asylum seeker	52	34.7
Immigrant	34	22.7
Marital status		
Never married	38	25.3
Married/partner	97	60.6
Divorced	2	1.3
Widowed	13	8.6
Exposure to traumatic events (AWES), mean (SD)	12.9 (3.3)	
Hopkins symptoms checklist (depression subscale only)^a^	22	14.7
Probable PTSD^b^	69	46

^a^ HSCL—25 ≥ 1.75

^b^ IES-R ≥ 34

### Clinical characteristics

Forty-six percent of participants met the threshold (≥34) for clinically significant PTSD symptomatology utilizing the IES-R. In comparison, reported rates of PTSD in NSMHWB ranged from 6.4 % for 12-month prevalence to 12.2 % for lifetime prevalence, which are between two to fivefold less than in our sample. Similarly, 14.7 % of our sample was scoring above the cut-off for probable depression (≥1.75) which is greater than both the 12-month and lifetime prevalence rates for depression reported in the NSMHWB of 4.1 and 11.6 %, respectively. The average number of traumatic events experienced by the refugee sample was 12.9 (SD = 3.3).

### Mental health literacy survey

In response to the question ‘What would you say is Mariam’s/Ahmad’s main problem?’ 46 respondents (30.7 %) chose PTSD, and a further 39 (26 %) chose fear. Moreover, 23 respondents (15.3 %) thought the character was suffering from depression, with stress being the next most commonly selected option by 19 respondents (12.7 %). Collectively, these responses accounted for 84.7 % of all responses. By way of comparison, 34.3 % of the general Australian public surveyed in the 2011 NSMHLS gave the correct psychiatric label of PSTD to the vignette.

Table 2 shows the percentage of respondents who considered interventions within each subcategory (treatment activities, medicines or people) as ‘helpful’, ‘harmful’ or ‘neither’ for the problem described. As can be seen, ‘improving diet or exercise’ was the treatment activity most often considered helpful (96.7 %) followed by ‘getting information about the problem’ and ‘psychotherapy focusing on past’ (both responses equally selected by 86.7 %). The single *most* helpful treatment activity selected was ‘finding new hobbies’ (18 %). By comparison, the top three helpful interventions selected by participants in the NSMHLS were ‘physical activity’ (93.5 %) ‘get out more’ (88 %), and ‘learn relaxation’ (87.9 %). With respect to medication, ‘vitamins and minerals’ were most commonly noted as helpful (76 %), with ‘herbal medicine’ seen to be the *most* helpful medication (33.3 %). This differs slightly from that reported in the NSMHLS, where ‘antidepressants’ (43.4 %) and ‘vitamins’ (36.6 %) were seen as helpful. In terms of assistance from people, the Afghan participants most frequently cited psychiatrists as being helpful (99.3 %), followed by psychologists (98.7 %) and GPs (90.7 %). In the NSMHLS, by comparison, the persons considered most likely to be helpful were counselors (91.3 %), GPs (85.6 %) and psychiatrists (84.2 %).

**Table 2:** Perceived helpfulness of interventions for PTSD among study participants (n = 150)

Interventions	Helpful	Harmful	Neither	Most helpful^a^
Treatments and activities				
Reading Koran or Bible	62	0	38.0	6
Finding new hobbies	86	0	14	18
Psychotherapy focusing on relationships with others	64	2.7	33.3	1.3
Prayer session	37.3	10.7	52	1.3
Improving diet or exercise	96.7	0	3.3	16
Psychotherapy focusing on past	86.7	3.3	10	14
Relaxation	63.3	2	34.7	2
Psychotherapy focusing on changing thoughts and behaviours	84	1.3	14.7	14.7
Getting information about problem	86.7	1.3	12.0	13.3
Reading a self-help book	32.7	10.7	32.7	0
Trying to deal with problem alone	7.3	76.7	16.0	0
Talking about problem	56.7	10.0	33.3	9.3
Admission to a psychiatric hospital	38	28.7	33.3	2.7
Traditional therapies	37.3	18	44.7	1.3
Hypnosis	23.3	8	68.7	0
Drinking alcohol to relax	0	12.7	87.3	0
Medications				
Vitamins and minerals	76	2	22	33.3
Herbal medicines	52.7	6.7	40.7	33.3
Antidepressants	40	13.3	46.7	20
Medication to help relax	32.7	37.3	30	13.3
People				
Psychiatrist	99.3	0	0.7	43.3
Family member	50.7	4	45.3	14
GP	90.7	0.7	8.7	15.3
Psychologist	98.7	0	1.3	14.7
Religious leader	30.7	11.3	58	4
Close female friend	42.7	4	53.3	2
Afghan social group/club	58	5.3	36.7	1.3
Close male friend	41.3	6.7	52	1.3
Community mental health worker	87.3	0.7	12	2.7
Community religious organization	41.3	8	50.7	1.3
Telephone counselling line	24.7	3.3	72	0

^a^ Percentage of sample rating the specific intervention item as ‘the most helpful’ for treating problem described in vignette

### Factors affecting response to particular questions

Participants who selected PTSD as the main problem had more years of education (8.28) than participants who selected fear as the main problem (4.41) (*p* = 0.010), whereas participants with fewer years of education were more likely to consider reading the Koran (rs = −0.267, p = 0.001) and using traditional therapies (rs = −0.272, p = 0.001) as helpful compared with participants with higher levels of education. Older participants were more likely to consider a prayer session (rs = 0.275, p = 0.001) and religious leader as helpful (rs = 0.389, p = 0.000) than younger participants. There were no other associations between demographic characteristics and responses to the MHL survey that were significant at the 0.01 level and no associations between clinical characteristics, namely, those with clinically significant levels of PTSD or depression symptomology and responses to the MHL survey that were significant at the 0.01 level.

## Discussion

This study sought to elucidate aspects of MHL relating to PTSD in a group of Australian based resettled Afghan refugees, namely, problem recognition and beliefs about the helpfulness of activities, treatments and treatment providers. Approximately one-third (30.7 %) of the participants identified the problem described in the vignette as PTSD. Improving diet or exercise, finding new hobbies, getting information about the problem and specific psychotherapy were the treatments most likely to be considered helpful for the problem described, whereas psychiatrists, psychologists and GPs were the treatment providers most likely to be considered helpful. Taking vitamins and minerals was also highly regarded, whereas the use of psychotropic medication was viewed less favorably. Participants with lower levels of education were less likely to identify the problem described as PTSD and both participants with lower levels of education and older participants were more likely to consider religious worship to be helpful than participants with higher levels of education and younger participants. There were no associations between participants’ levels of PTSD and depressive symptomatology and the aspects of MHL addressed.

Of note is that the proportion of participants in the current study who identified the vignette as describing a person with PTSD was similar to, although slightly lower than, that of the general Australian population (30.7 versus 34.3 %) [15]. While this finding is encouraging, particularly when compared with the very low rates of identification of PTSD (14.2 %) observed in our previous study of Iraqi refugees resettled in Australia [16], it is still far from ideal. Further, whereas almost half (46 %) of participants in the current study had clinically significant levels of PTSD, identification among this sub-group was no better than that observed among asymptomatic participants. If less than one-third of individuals with clinically significant symptoms of PTSD recognise that they have this problem then it is not surprising to find low uptake of mental health services in this sub-group [12]. The finding that participants with higher levels of education were more likely to identify the problem described as PTSD than those with lower levels of education may reflect an association between higher levels of education and greater exposure—and receptiveness—to western, biomedical models of mental health problems and their treatment.

The finding that self-help interventions, such as improving diet and exercise, finding new hobbies, and getting information about the problem, were highly regarded in the treatment of PTSD among participants in the current study is consistent with findings from studies of MHL relating to a broad range of mental health problems in various populations, including the Australian NSMHLS [15]. In the current study, this finding may reflect not just a universal preference for the use of less confronting and less potentially stigmatising interventions, but also an expression of the perceived importance of “keeping oneself busy”, this being a coping mechanism known historically to be favoured in Afghan populations [26]. At the same time, participants in the current study viewed the use of specific psychotherapy in the treatment of PTSD favourably. The vast majority considered both “psychotherapy focusing on changing thoughts and behaviours” (84 %) and “psychotherapy focusing on the past” (86.7 %) to be helpful. In this respect the current study participants’ views are consistent with “evidence-based practice”, in which the use of specific psychotherapy, along with that of anti-depressant medication, is advocated in the treatment of PTSD [27].

The preference for the use of “vitamins and minerals” and “herbal medicine” over the use of anti-depressant and/or anxiolytic medication in the treatment of PTSD observed among participants in the current study is also consistent with findings from previous studies of MHL relating to various mental health problems observed in various study populations [13], although participants in the Australian NSMHLS regarded the use of anti-depressant medication more favorably than participants in the current study. Evidence suggests that interventions and ceremonial rituals, such as spiritual leaders reading the Koran or praying with the person who is unwell, are readily available and acceptable in cross-cultural communities, while medications such as antidepressants were said to worsen the symptoms of mental illness in at least one study of (Somali) refugees [28]. In the current study, 62 % of participants reported reading the Koran and 37.3 % reported believing that that prayer sessions would be helpful for the problem described. Consistent with this finding, Miller and colleagues (2009) [29] noted that faith in God and prayer are among the ways Afghans report coping with mental illness.

Also of interest is the finding that participants in the current study considered psychiatrists, along with psychologists and general practitioners, to be the ‘treatment providers’ most likely to be helpful. This was comparable with participants in the Iraqi refugee study where 84.5 % selected seeking help from a psychiatrist as most helpful [16]. Both the Iraqi and Afghan studies showed variations from the preferred treatment providers noted in the NSMHLS, where counselors followed by GPs were most helpful in treating PTSD [15]. The reason for these differences warrants further examination, as there may be social or cultural elements surrounding the role of psychiatrists that are particular to the Iraqi and Afghan populations. It also is possible that PTSD is perceived among individuals in these cultures as being a more severe “brain-based” illness than certain other mental health problems, such that treatment from a mental health professional who is also a physician is warranted. Finally, another reason for the higher endorsement of psychiatrists observed among participants in the current study may not only resonate from the trust they place in the biomedical community, but it also is possible that this endorsement reflects, in part, an artifact of social desirability in responses.

Finally, it is notable that a majority of participants in the current study (76.7 %) believed that ‘dealing with the problem alone’ would be harmful. This is in contrast to the Iraqi study, where more than half of the participants believed that trying to deal with problems alone is possibly helpful [16]. Hence, Afghan refugees appear to recognise that help from some source at least is important when dealing with the symptoms of PTSD, whereas dealing with the problem in isolation may be considered to be beneficial—or necessary perhaps—among certain other refugee populations.

These differences and similarities across two refugee populations in resettled in Australia provide useful insights for treating professionals particularly in the delivery of mental health education and promotion and highlight the need for studies of MHL to be conducted with specific communities of interest at the local level rather than attempting to generalise from studies of the mainstream community or from the same cultural group in a different parts of the world. More generally, differences in treatment preferences suggest different ways in which clinicians may need to respond to and work with refugee communities if they wish to achieve true therapeutic alliance. This can be done through close collaborative efforts with targeted refugees in which the incorporation of aspects of culture, such as the importance of prayer, can be weaved into the development of intervention programs [30], in addition to seeking the support and input of community elders [31]. This then needs to be dovetailed with better informed treating professionals, proficient in understanding the unique traumatic background of differing groups of refugees. Furthermore, greater consideration will need to be given to understanding post-resettlement stressors at the local level in development of such programs. Related issues concern the relative importance of addressing PTSD and related symptomatology in resettled refugee populations and addressing individuals’ immediate needs. It may be that, depending of where the refugee is in their resettlement journey, addressing the immediate expressed needs of refugees and providing opportunities for the establishment of social capital and resiliency, may be more appropriate than a Western biomedical approach to their mental health care [6, 32]. In future research of this kind it would helpful to assess both aspects of MHL deemed to be pertinent and sources of post-resettlement stress, so that the relative importance of these variables in informing programs aimed at reducing the impact of posttraumatic stress can be examined.

Strengths of the current study included the relatively large sample size, which permitted analysis of the associations between MHL, clinical characteristics and socio-demographic characteristics and the inclusion of measures of both PTSD and depression as well as exposure to trauma. Administration of the survey instrument via in-person interviews and the recruitment of participants by a researcher who could speak the language, and was of the same nationality as participants, helped to establish rapport and trust and thereby improved the recruitment process and the quality of the data collected.

A limitation of the current study is the use of self-report measures of mental health. Additionally participants were volunteers from within the resettled Afghan community, thus, the self-selected convenience sample may have introduced bias. However, it should be noted that a lack of a clear sampling frame and limitations with Census data hinders true assessment of representativeness of such difficult-to-reach populations [33]. Hence, the generalizability of the current findings to the total population of Afghan refugees residing in Adelaide is unclear. For example, it is possible that more socially engaged and connected individuals were over-represented among study participants, given the recruitment methods employed. If so, then responses to certain items of the MHL survey may have been more optimistic and/or informed than would be the case in a more representative sample. On the other hand, participants in the current study had markedly elevated levels of PTSD symptomatology relative to the general Australian population, and study representativeness was not considered to be paramount for an initial study of this kind in a difficult-to-access population. Future research with this population should include assessment of other key variables, such as actual help seeking and post-resettlement stressors, as mentioned above.

In conclusion, the current study suggests Afghan refugees resettled in Australia are likely to have very high levels of distress and PTSD symptomology. Of greater concern, the study revealed a poor understanding of mental health symptoms with large numbers of participants not seeking treatment deemed appropriate for their mental illness, based on the vignettes. The results of this study demonstrate differences in terms of MHL and treatment practices between the Afghan population and both mainstream and Iraqi populations. This indicates the need for specific and targeted mental health services and treatment plans, which recognise cultural differences and values in order to bridge the gap between Western medical models for treatment and Afghan refugee treatment preferences.

## Additional file

10.1186/s13033-016-0065-7The vignette used in the mental health literacy survey.

## Abbreviations

PTE: potentially traumatic events
PTSD: post-traumatic stress disorder
MHL: mental health literacy
AWES: Afghan War Experience Scale
IES-R: the impact of events scale-revised
HSCL–25: Hopkins Symptoms Checklist–25
NSMHWB: Australian National Survey Of Mental Health And Wellbeing
NSMHLS: National survey of Mental Health Literacy and stigma
