# Supplementary material for: Beliefs and knowledge about post-traumatic stress disorder amongst resettled Afghan refugees in Australia
Source: Int J Ment Health Syst. 2016 Apr 12;10:31. doi: 10.1186/s13033-016-0065-7 (PMC4828823; doi:10.1186/s13033-016-0065-7)
Supplement: Supplementary file 1 — 10.1186/s13033-016-0065-7The vignette used in the mental health literacy survey. [file 13033_2016_65_MOESM1_ESM.docx]

**Additional file**

Additional file 1 – **The vignette used in the mental health literacy survey**

Mariam* is a 37 year old married woman with 3 children, a daughter aged 7, and two sons, aged 5 and 3. Mariam has been living in Australia for the past year and has attended her local GP on several occasions with the primary complaint of an inability to sleep. The problem with her sleep started just before she left her homeland, Afghanistan, four years ago. Prior to leaving Afghanistan, Mariam was kidnapped by insurgents and held captive until her husband paid the demanded ransom. During her kidnapping, Mariam was beaten and threatened with death. She reports that during the kidnapping she felt intense fear and helpless. She reports constant nightmares in which images of death, killing and being kidnapped by masked men disturb her sleep. She avoids talking about her attack and watching Afghan news channels in case there is a story about people being kidnapped and murdered. She is easily startled when she hears loud sounds such as a car backfiring or fireworks. She has very little interest in things around her, including her children's lives and feels little affection towards them. Finally, when questioned on how she views her future and plans for her life, Mariam replies that she does not have a future and doesn't believe she will live a long life.

*Replaced with Ahmad in the case of male participants
